# The road to a polio-free Uganda; contribution of the Expanded Program on Immunization Laboratory (EPI-LAB) at Uganda Virus Research Institute

**DOI:** 10.4314/ahs.v23i3.23

**Published:** 2023-09

**Authors:** Mary B Nanteza, Phionah Tushabe, Henry Bukenya, Prossy Namuwulya, Theopista Kabaliisa, Molly Birungi, Mayi Tibanagwa, Immaculate Ampeire, Proscovia Kakooza, Edson Katushabe, Josephine Bwogi, Barnabas Bakamutumaho, Miriam Nanyunja, Charles R Byabamazima

**Affiliations:** 1 Uganda Virus Research Institute, Plot 51-59 Nakiwogo Road, P. O. Box 49, Entebbe, Uganda; 2 Ministry of Health, Government of Uganda, Plot 6, Lourdel Road, Nakasero P. O. Box 7272, Kampala, Uganda; 3 World Health Organization Office, Plot 60 Prince Charles Avenue, Kololo, Kampala; 4 World Health Organization AFRO, East and Southern Africa (ESA), Nairobi, 45335 Nairobi, Kenya; 5 World Health Organization AFRO, East and Southern Africa (ESA), Harare, 82-86 Enterprise Road, Highlands, P. O. Box BE 773, Belvedere, Harare, Zimbabwe

**Keywords:** Poliovirus, eradication, acute flaccid paralysis, laboratory surveillance, Uganda

## Abstract

**Background:**

The control of poliomyelitis in Uganda dates back as far as 1950 and acute flaccid paralysis (AFP) surveillance has since been used as a criterion for identifying wild polioviruses. Poliovirus isolation was initially pursued through collaborative research however, in 1993, the Expanded Program on Immunization Laboratory (EPI-LAB) was established as a member of the Global Poliovirus Laboratory Network (GPLN) and spearheaded this activity at Uganda Virus Research Institute.

**Objectives:**

The aim of this report is to document the progress and impact of the EPI-LAB on poliovirus eradication in Uganda.

**Methods:**

Poliovirus detection and identification were achieved fundamentally through tissue culture and intra-typic differentiation of the poliovirus based on the real-time reverse transcriptase polymerase chain reaction (rRT PCR). The data obtained was entered into the national AFP database and analysed using EpiInfo^TM^ statistical software.

**Results:**

Quantitative and qualitative detection of wild and Sabin polioviruses corresponded with the polio campaigns. The WHO target indicators for AFP surveillance were achieved essentially throughout the study period.

**Conclusion:**

Virological tracking coupled with attaining standard AFP surveillance indicators has been pivotal in achieving and maintaining the national wild polio-free status. Laboratory surveillance remains key in informing the certification process of polio eradication.

## Introduction

The World Health Assembly of 1988 committed member states to the global eradication of poliomyelitis 1. Successive global polio eradication deadlines have been re-adjusted since the year 2000 2-4 and it is expected that from 2023 endemic poliovirus transmission will cease forever across all parts of the world 5. Prior to global commitment to eradicate polio, AFP surveillance for poliovirus search was documented in Uganda. From 1950 to 1951 the East African Virus Research Institution (EAVRI) in collaboration with Poliomyelitis Research Center of Baltimore USA led the poliomyelitis control initiative. Later, an alliance was established between EAVRI and World Health Organization (WHO).

In 1977, the continuity of AFP surveillance was interrupted by the break-up of the East African Community (EAC) under which the EAVRI operated. This resulted in the emergence of the current Uganda Virus Research Institute (UVRI). Work on polio isolation halted until 1993 when the Uganda National Expanded Program on Immunization - Laboratory (EPI-LAB) became functional at UVRI as part of the efforts towards global polio eradication. The laboratory became a member of the global poliovirus laboratory network (GPLN) 6 and then, a member of the Africa Polio laboratory network 7,8.

The UVRI EPI Laboratory had institutional memory and infrastructure from the WHO virus study team that had worked on polio and non-polio enteroviruses when the Institute was still serving as the EAVRI 9. Initially, progress of virological surveillance in support of the PEI was slow 10 but improved by 1997 11. To sustain the expanding scope of AFP surveillance, the EPI Laboratory developed a technical collaboration with a better-resourced HIV/AIDS/STDs surveillance unit of Ministry of Health (MOH) 12,13. With time, the Uganda National Expanded Program on Immunization (UNEPI) contributed to the “polio weekly active surveillance search” a collaboration site that was established at “Mulago Round Table Polio clinic”. This site was a key data source on past clinical polio cases and specimens from suspected polio patients.

From 1996, collaboration was progressively extended to Ministry of Health Disease surveillance and Health Management and Information System (HMIS). In the process of strengthening AFP surveillance, the EPI Lab contributed to building the foundation and establishment of integrated disease surveillance in Uganda. There was increasingly strengthened collaboration with WHO while progressively reducing the dependence on the HIV/AIDS/STDs surveillance unit. Since 1996, the laboratory was designated by WHO as an Inter-Country Polio Laboratory and has provided support to other countries in the sub-region that did not have national polio laboratories. In Uganda, the last indigenous wild poliovirus was detected in 1996 though importations were detected in 2009 and 2010 14,15. Absence of indigenous wild poliovirus isolation in Uganda 13 in the presence of quality AFP surveillance led the “National Polio Certification Committee” to submit a Uganda Polio-Free documentation and request for endorsement from the African Regional Certification Commission (ARCC). The ARCC reviewed the document and certified Uganda free of wild poliovirus in October 2006 16.

In accordance with the recommendations by the ARCC, post-certification laboratory-backed AFP surveillance continued in Uganda. The laboratory was further able to detect polioviruses from vaccine-associated paralytic poliomyelitis (VAPP) cases and type 2 ambiguous Vaccine-Derived Poliovirus (aVDPV) strains 17,18 that respectively supported the switch from trivalent oral polio vaccine (tOPV) to bivalent OPV (bOPV) and led to the initiation of environmental surveillance to reinforce AFP surveillance in Uganda.

## Methods

The methods used along the journey to a polio-free Uganda are described from 1993 till August 2020 when Africa was certified free of indigenous wild poliovirus 19.

### Sample collection and transportation

Following the identification of a suspected AFP case, two stool specimens were collected 24 - 48 hours apart using the WHO AFP surveillance guidelines and transported in specimen carriers under reverse cold chain to the EPI Laboratory at UVRI. Specimens were received aseptically by trained personnel, assessed for stool condition, and kept at 4-8^o^C if these were to be tested within 72 hours or kept at -20^0^C for longer term storage. Virus extraction from specimens was performed using chloroform treatment in keeping with standard protocols defined in the WHO laboratory manual 20.

### Virus isolation and intra-typic differentiation

From 1993 until 2000, virus isolation was performed using Hep-2C cell line and RD cell line and results were expected 28 days post specimen receipt. Virus isolation on Hep-2C and RD cell lines was not selective between polio and non-polio enteroviruses. Appropriate serum neutralizing antisera in tissue culture was then introduced for typing adapting a method initially described by researchers at the previous Central Public Health Laboratories, Colindale, UK 21 before the setup of the Global Polio Laboratory network.

After 2000, the polio laboratory network switched from use of Hep-2C to a more polio specific cell line; L20B 22 supplied by the CDC Laboratory in Atlanta. Virus isolation was performed on L20B and RD cell lines. The viruses growing on RD cells would be passed on to the polio selective L20B cell line. The L20B positive cultures were since then labeled “Suspected poliovirus isolates”. The introduction of L20B cells led to a new and shorter virus isolation algorithm that eliminated the need for virus neutralization step to identify polioviruses and non-polio enteroviruses.

Polio isolates were then referred to the National Institute of Virology, South Africa for Intra-typic Differentiation (ITD) and would group the viruses into wild and/or Sabin polioviruses and assigned respective serotypes. The first ITD method comprised of an Enzyme Linked Immunosorbent Assay (ELISA) and Polymerase Chain Reaction (PCR) method which had to be concordant for the final definitive result.

In 2010, real time RT-PCR was introduced as a replacement of the ELISA ITD and conventional PCR to a more sensitive and faster ITD method in the network. Its development was spearheaded by the Global Specialized CDC Laboratory, Atlanta. It is still used for virus identification and has an aspect that screens for Vaccine Derived Polioviruses (VDPVs). Intra-typic differentiation / VDPV screening kit versions have changed over the years in a bid to improve the methods for enhanced sensitivity, specificity and timeliness 23–25, and to address the changing needs of the advanced PEI.

### Data collection and analysis

Between 1993 and 1997 the EPI Laboratory in close collaboration with UNEPI and the Ministry of Health was able to train over 600 health workers across the country. This process led to the start of AFP case-based laboratory supported surveillance and thus generation of data. The AFP laboratory data was entered in an Access database developed by WHO on the CDC EpiIfo™ platform, cleaned and harmonized with the WHO AFP surveillance database to eliminate discrepancies and improve quality.

### Quality assurance

Quality laboratory performance was based on regular monitoring of adherence to standard operating procedures (SOPs), annual accreditation reviews by the World Health Organization and the external WHO proficiency panel testing of unknown specimens 26. Quantitative and qualitative indicators were used to promote quality control (QC) and quality assurance (QA) measures at the laboratory level.

## Results

**(a).** The pattern of age and sex among AFP cases investigated is represented in the table 1 below.

**Table 1:** The sex and age distribution versus AFP incidence in Uganda from 1994 to August 2020

Age in months	Males (%)	Females (%)	Total AFPs
0 - 9	174 (52)	160 (48)	334
10 - 19	723 (58.4)	514 (41.6)	1237
20 - 29	623 (57.5)	461 (42.5)	1084
30 - 39	470 (59.3)	322 (30.7)	792
40 - 49	353 (58.8)	247 (41.2)	600
50 - 59	187 (61.1)	119 (38.9)	306
60	133 (58.8)	93 (41.2)	226
>60	1253 (58.2)	901 (41.8)	2154
Age missing	1128 (58.3)	807 (41.7)	1935
**Total**	5044 (58.2	3624 (41.8)	8668

**Legend:** The investigation has been conducted largely among the ≤ 5 years of age. Percentage (%) refers to the proportion of males and females for a specified age interval and ‘age missing’ indicates that the ages of the children were not specified.

The number of AFP cases was highest at the age of 10 - 19 months (14.3%). Acute flaccid paralysis was more common in males (58%) than females (42%) (p<0.001) (pre-testing in STAT14). The difference was highest among children 50-59 months of age.

**Figure 1:**
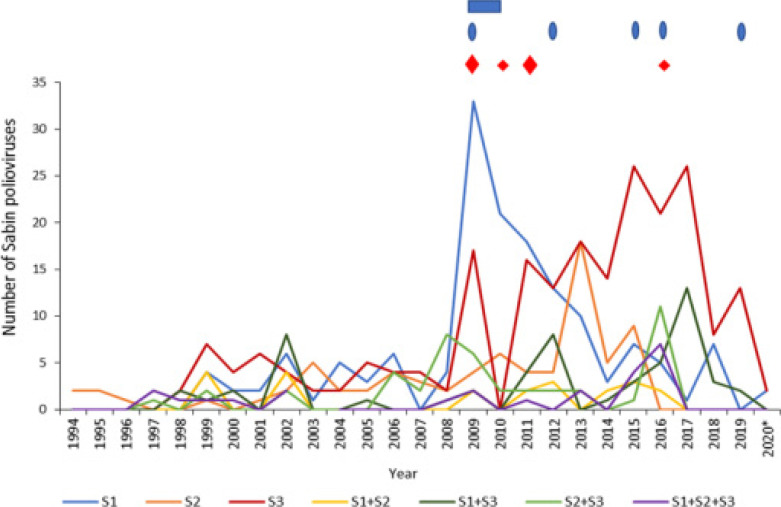
The variation of Sabin poliovirus secretion in Uganda from 1994 to August 2020 **Legend:** Abbreviations, NIDs - national immunization days, SNIDs - sub-national immunization days, 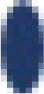-NIDs conducted, 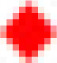 -2 rounds of SNIDs, 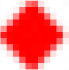 -5 rounds of SNIDs, 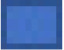-WPV1 importation, S-Sabin poliovirus, and the symbol “*” refers to data available as of August 2020. The data available on supplemental immunization activities starts from 2009; and aggregated data has been used for Sabin poliovirus secretion.

The laboratory has detected wild and Sabin polioviruses in stool from AFP cases and later in sewage (2017). Variation in the Sabin poliovirus secretion from AFP cases between 1994 and 2020 is shown in figure 1.

The detection of Sabin polioviruses was evident from 1994 and thereafter stabilized till 2008 with no unique pattern for the 3 serotypes. In 2009, there was predominant secretion of Sabin poliovirus 1; Sabin poliovirus 2 secretion increased in 2013 and stopped in 2016. Overall, there was predominant secretion of Sabin poliovirus 3 from 2014 to 2019. Although the detection of Sabin polioviruses declined in 2014, Sabin poliovirus 1 and 3 were the only co-circulating strains since 2017 and decreased in 2020. Sabin poliovirus serotype 2 has not been detected in Uganda since 2017 to 2020.

**Figure 2:**
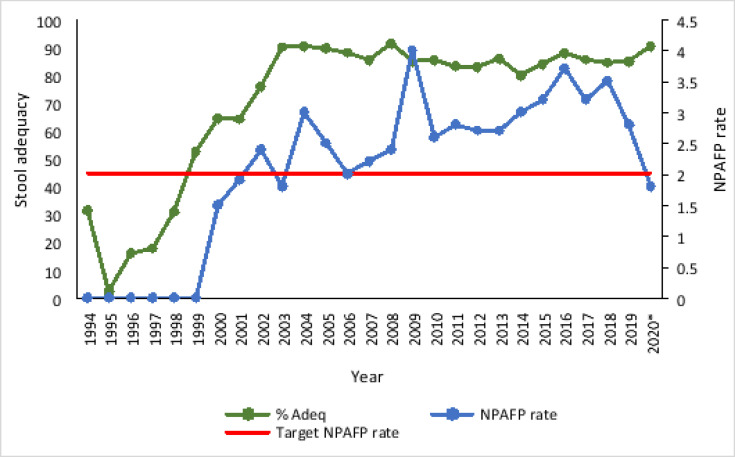
Stool adequacy versus non-Polio AFP rate in Uganda from 1994 to August 2020 **Legend:** The NPAFP rate stands for the non polio AFP rate and is defined as the number of AFP cases detected in 100,000 children below 15 years of age. The horizontal line shows the adopted standard NPAFP rate of 2.0 for Uganda. Stool adequacy is described as the collection of two stool specimens 24 - 48hrs apart within 14 days of the onset of paralysis. “%Adeq” refers to the proportion of qualifying specimens collected compared to the total number of specimens collected per year. The symbol “*” indicates data available as of August 2020

**(b).** A graph of surveillance indicators namely stool adequacy and non-polio-AFP (NPAFP) rate is shown in Figure 2.

Stool adequacy improved earlier than the NPAFP rate. The NPAFP rate increased from 1999 and markedly raised in 2002, 2004, 2009, 2016, and 2018 before dropping in 2020. From 2006 to 2019 the NPAFP rate was consistently above the standard NPAFP cut off of 2.0 adopted for the national AFP surveillance system. It ranged from 2.3 to 4.0 for the specified period 27. The stool adequacy was consistently above the MOH/ WHO recommended minimum target of 80% between 2003 and 2020.

**Figure 3:**
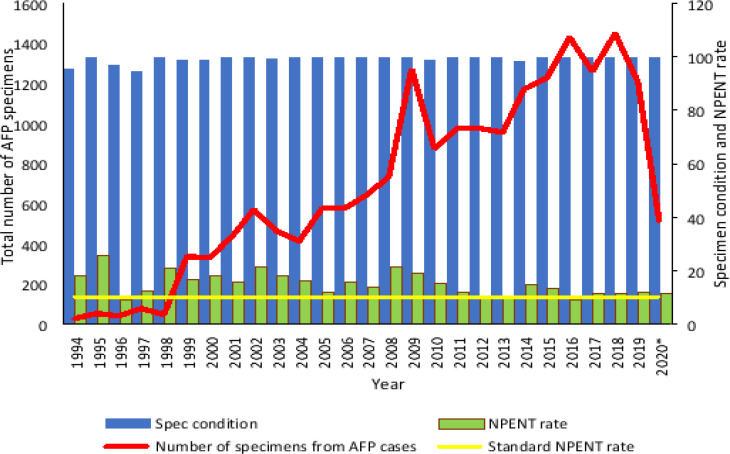
The trend of the total number of AFP specimens, specimen condition and non polio enterovirus isolation rate in Uganda from 1994 to August 2020 **Legend:** The term “NPENT” represents non polio enterovirus and the NPENT rate refers to the proportion of NPENTs detected compared to the total number of specimens cultured per year. The horizontal line shows the minimum target NPENT rate of 10% according to the MOH/ WHO guidelines. The target for good specimen condition is ≥80% and it refers to the proportion of specimens delivered in good condition when compared to the total number of stool specimens of AFP cases received per year. The symbol “*” indicates data available as of August 2020

**(c).** Figure 3 shows the total number of AFP specimens, specimens' condition, and NPENT isolation rate in Uganda.

The specimen condition and NPENT rate were consistently above the minimum standard targets of 80% and 10% respectively from 1994 to 2020 except in 1996 and 2016 when the NPENT rate was below the target. The number of AFP specimens gradually increased from 1998 to 2018 with peaks in 2002, 2009, 2016 and 2018.

**Figure 4:**
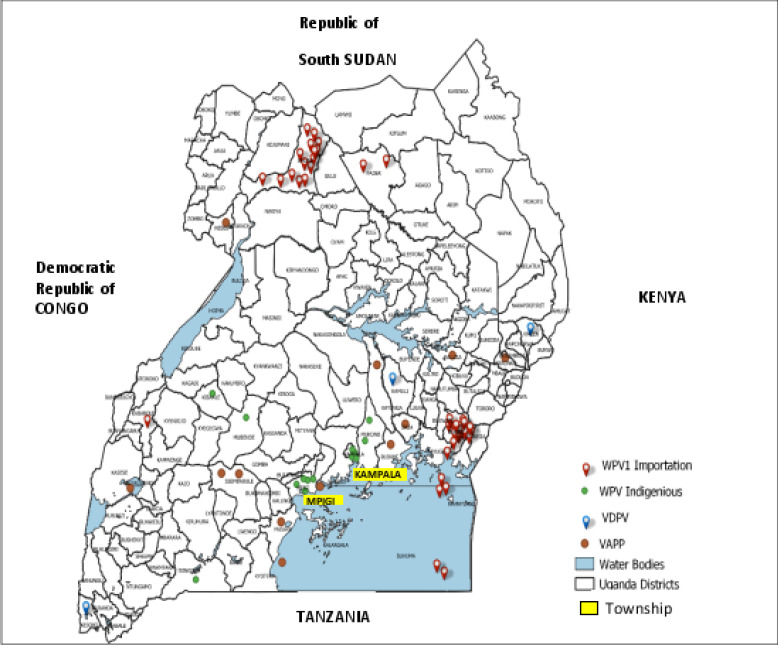
The distribution of WPV serotype 1, VDPV serotype 2, and Sabin serotype 1, 2, and 3 among VAPP cases in Uganda (1994-2020) **Legend:** WPV: Wild poliovirus; VAPP: Vaccine -associated paralytic poliomyelitis; VDPV: Vaccine-derived poliovirus; xxx: the district was not captured; ( ): number of times WPV1 has been detected from the district *: WPV1 importation; [SL1]: Sabin-like poliovirus type 1, [SL2]: Sabin-like poliovirus type 2, and [SL3]: Sabin-like poliovirus type 3

**(d).** The laboratory detected wild polioviruses (WPVs), vaccine-derived polioviruses (VDPVs) and Sabin polioviruses from cases of vaccine associated paralytic poliomyelitis (VAPP). Figure 4 shows the distribution of WPV serotype 1, VDPVs serotype 2, and Sabin serotype 1, 2, and 3 detected from VAPP cases between 1994 and 2020.

Table 2 summarizes the number of WPV, VAPP and VDPV cases by serotype by year.

**Table 2:** Polio serotypes detected between 1994 and 2020 in Uganda

Poliovirus type	Year	Number of cases and contacts	District(s)
WPV 1	1994	3	xxx, Kampala (2)
	1995	6	Kibaale, Iganga, Mubende, Mpigi (2) Kampala
	1996	7	Mpigi (3), Kabarole, Kampala, Mukono (2)
	2009*	10	Amuru (9), Pader
	2010*	12	Bugiri (9), Mayuge (3)
VAPP	2007	2	Bukwo [SL3], and Kayunga [SL2 & 3],
	2008	1	Pallisa [SL2 & 3]
	2009	5	Nebbi [SL3], Masaka [SL2], Masaka [SL1], Sembabule [SL1], and Kasese [SL3]
	2010	1	Sironko [SL2&3]
	2011	2	Jinja [SL1], and Wakiso [SL2&3]
VDPV2	2014	3	Kamuli, Kween, and Kisoro

**Legend:** WPV: Wild poliovirus; VAPP: Vaccine -associated paralytic poliomyelitis; VDPV: Vaccine-derived poliovirus; xxx: the district was not captured; ( ): number of times WPV1 has been detected from the district

* WPV1 importation; [SL1]: Sabin-like poliovirus type 1, [SL2]: Sabin-like poliovirus type 2, and [SL3]: Sabin-like poliovirus type 3

Indigenous wild polio serotype 1 viruses detected between 1994 and 1996 were confined at two townships that included Kampala, the capital city of Uganda. WPV1 importation occurred in 2009 in Amuru district, along the major northern route from the Republic of South Sudan to Uganda. Subsequent WPV1 importation was detected in the following year (2010) in Bugiri district located in the eastern part of the country alongside the border of Kenya. In both instances, the viruses were confined largely to the reporting districts. The VAPP cases were identified largely in the southern part of the country.

### Annual Proficiency and Accreditation Performances

The proficiency and accreditation evaluations stipulated by WHO/MOH were consistently above the minimum target of 90% except during the initial 2 years (1997 and 1998) when polio eradication activities were implemented in the country.

## Discussion

The pattern of sex and age distribution versus AFP incidence was performed largely in the under 60 months old children because it is the target age group for routine and supplementary polio immunization activities. Acute flaccid paralysis was more common in males than females (p<0.001). The increased incidence of AFP observed in males could be attributed to the tendency of males being more outdoorsy compared to females hence making them more prone to getting into contact with the virus. The highest incidence of AFP cases was observed in children aged 10-19 months old which is outside the routine immunisation age bracket. It appears supplementary immunisation might have a role to play on the incidence of AFP however, mothers tend not to honor immunization appointments 28 and the many AFP cases observed could be a result of overlapping dozes of immunization. It is also noted that the age of 22% (1935/8668) of AFP cases was not registered and this might have affected the correlation of age and incidence of AFP.

Sabin secretion became stable after the establishment of the EPI Laboratory in 1997 and increased secretion was noted in the later years. The increase in Sabin secretion corresponded with the immunization activities. In 2009, the marked increase in Sabin poliovirus 1 secretion was a result of massive polio campaigns that were implemented using monovalent OPV1 in response to WPV type 1 importation. In 2016, OPV2 was withdrawn and tOPV (Sabin poliovirus 1, 2, and 3) was replaced with bOPV (Sabin poliovirus 1 and 3). Polio campaigns were performed using tOPV to boost the OPV2 population immunity prior to the switch and furthermore to reinforce population immunity in high-risk areas like the poorly performing districts and districts at border regions 29–31. An increase in mixtures of Sabin polioviruses was observed. Largely, there was preferential secretion of Sabin poliovirus 3. Sabin 3 has been reported to have a better replicative fitness than Sabin poliovirus 1 and 2 32.

In 2014, there were no NIDs nor SNIDs activities and Sabin secretion decreased for the three serotypes. After the withdrawal of OPV2, cessation of Sabin poliovirus 2 secretion was observed in 2016. A decline of Sabin poliovirus secretion 1 and 3 was also observed in 2020 and coincided with the period when immunisation and surveillance activities were deterred by the COVID-19 pandemic. In addition, a partial year “study period of observation” was considered (January to August 2020).

Stool adequacy improved from 1995 and remained stable above 80% from 2003 whereas the NPAFP rate lagged behind and improved from 2000 with peaks in years with polio campaigns. The challenge then was the inaccurate reporting of data collected from the health facilities 10 consistent with greater need for resources & training of clinicians and surveillance teams. The standard NPAFP rate for detecting suspected cases of poliomyelitis is 1.0 - 2.0 33. In Uganda, the upper limit of a NPAFP rate was adopted to increase the sensitivity of detecting AFP cases and ensure that no indigenous wild poliovirus nor VDPV remains in the country.

The NPENT rate was consistently above the minimum target throughout the study period except for a couple of years. Specimen condition which reflects on the integrity of the specimens collected was consistently above the minimum target of 80% implying that the viability of the polioviruses was preserved, and it is unlikely that any polioviruses went undetected on culture. In general, the trend of “NPAFP rate”, and “stool adequacy” improved with time and that of “specimen condition” and “NPENT rate” remained adequate. The stable increase of AFP specimens suggests improved AFP surveillance permitting exhaustive detection of polioviruses.

The imported wild polioviruses detected were well controlled in the districts of identification. All VDPVs described were ambiguous VDPVs. The VAPP cases detected from 2003 to 2011 were identified predominantly in the southern part of the country possibly because surveillance activities in the northern part of the country were halted by political insurgence. The proficiency and accreditation assessments for virus isolation and polio intratypic differentiation were met early in the journey for polio eradication in Uganda. The UVRI EPI Laboratory progressed from partial accreditation by WHO in 1997 to full accreditation status in 1999 and became one of the earliest laboratories in the East African sub-region to be fully accredited. The accreditation status was maintained for the rest of the study period.

The national EPI Laboratory has ably detected and monitored Sabin and wild polioviruses secretion and has contributed to the certification of wild poliovirus interruption in Uganda and the region of Africa. The collaborative approach between the HIV/ AIDS and EPI Laboratory surveillance units of Ministry of Health at the set-up of the laboratory sparked the development of the national integrated disease surveillance system. Such approach could be simulated for addressing related health priorities in similar settings.

## Data Availability

The data is available at the national and WHO AFP database
